# Colonic diaphragm disease without significant non-steroidal anti-inflammatory drug use: a case report

**DOI:** 10.1186/1757-1626-1-247

**Published:** 2008-10-17

**Authors:** Jo C Roche, Gareth Morris-Stiff, Carrie Champ, Geraint T Williams, Michael H Lewis

**Affiliations:** 1 Department of Pathology, Royal Glamorgan Hospital, Ynysmaerdy, Llantrisant, UK; 2 Department of Surgery, Royal Glamorgan Hospital, Ynysmaerdy, Llantrisant, UK; 3 Department of Pathology, University Hospital of Wales, Heath Park, Cardiff, UK

## Abstract

**Introduction:**

Colonic diaphragm disease is an uncommon condition usually associated with the long-term use of non-steroidal anti-inflammatory drugs.

**Case presentation:**

A 48-year-old woman presented as an emergency patient with abdominal pain and vomiting. Past medical history included inflammatory bowel disease of ulcerative colitis type for which she was taking azathioprine and prednisolone. On examination, she was shocked with signs of peritonism. Following resuscitation, she was taken for a laparotomy upon which a small amount of turbid fluid was identified but there was no direct evidence of an intra-abdominal perforation. A peritoneal lavage was performed and she was taken to the intensive care unit. A repeat laparotomy was performed on the sixth postoperative day, following a clinical deterioration and again no leak was identified. Given the history of ulcerative colitis, the perforation was presumed to be of colonic origin and a total colectomy and ileostomy was performed. Histopathological examination of the colectomy specimen revealed extensive colonic diaphragm disease with 30 thin-walled diaphragms, one of which reduced the lumen to a pin-hole. No perforation was identified.

**Conclusion:**

To the best of the our knowledge, this is the first report of the development of colonic diaphragm disease in the absence of a history of non-steroidal anti-inflammatory drug ingestion. Given the history of ulcerative colitis we believe that the disease may have arisen as a result of the healing of the underlying inflamed colon rather than as a direct effect of non-steroidal anti-inflammatory drugs.

## Introduction

Colonic diaphragm disease is an uncommon condition and when identified is usually related to long-term non-steroidal anti-inflammatory drug (NSAID) therapy, either prescribed or self-medicated [1]. We present a case of colonic diaphragm disease in a 48-year-old woman with a 4-year history of inflammatory bowel disease and no significant history of NSAID usage.

## Case presentation

A 48-year-old woman presented as an emergency on the surgical intake with a 24-hour history of severe generalised abdominal pain. She had vomited once prior to admission but there had been no alteration in bowel habit. No other symptoms were reported. She had a 4-year history of inflammatory bowel disease of ulcerative colitis type for which she was being prescribed azathioprine (50 mg/day) and prednisolone (5 mg/day).

On examination, she was shocked with a heart rate of 100 beats/minute, a blood pressure of 70/55, pyrexia of 38.2°C, a respiratory rate of 25 breaths/minute and oxygen saturation levels on air of 90%. Her abdomen was distended and diffusely tender with signs of peritonism. She was aggressively resuscitated. Her full blood count showed a neutropaenia of 3.6 × 10^9^/litre. An erect chest radiograph demonstrated free gas under the left hemidiaphragm. She was taken for a laparotomy at which point a small amount of turbid fluid was identified but there was no direct evidence of an intra-abdominal perforation. A peritoneal lavage was performed and she was taken to the intensive care unit.

On the sixth postoperative day, her condition deteriorated and she was returned to the theatre for a second laparotomy. Again, there was turbid fluid but no obvious perforation was identified. Methylene blue was instilled via a nasogastric tube to try and delineate a perforation of the foregut. However, as no leak was seen and since she had a history of colitis, it was presumed that the perforation must have been somewhere in the colon and so a total colectomy and ileostomy were performed. After which, she was returned to the intensive care unit for ventilatory and cardiovascular support. Her condition deteriorated and blood cultures following the operation revealed the presence of Gram-positive cocci. She made little postoperative improvement and had a cardiac arrest and died 7 days after the second laparotomy.

**Figure 1:**
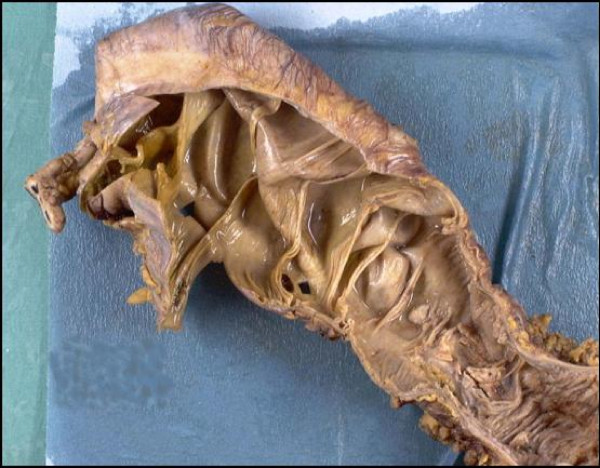
Macroscopic view of a resection specimen demonstrating numerous colonic diaphragms.

Histopathological examination of the gross colectomy specimen showed impacted hard faeces together with inspissated pale mucus. There was a fibrinous material covering much of the peritoneal surface. No obvious perforation site was seen but numerous 'diaphragms' were noted, most marked within the caecum. There were approximately 30 thin-walled diaphragms, 10 of which were complete (Figure 1). The diaphragm within the more distal part of the bowel reduced the lumen to a pin-hole. The mucosa in the area of the diaphragms appeared flattened but otherwise there were no signs macroscopically of active inflammatory bowel disease.

**Figure 2:**
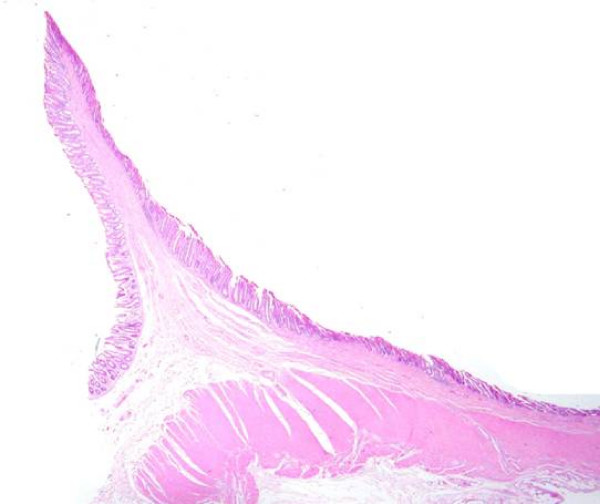
Microscopic examination of a specimen demonstrating elongated plicae (diaphragms) with submucosal fibrosis.

**Figure 3:**
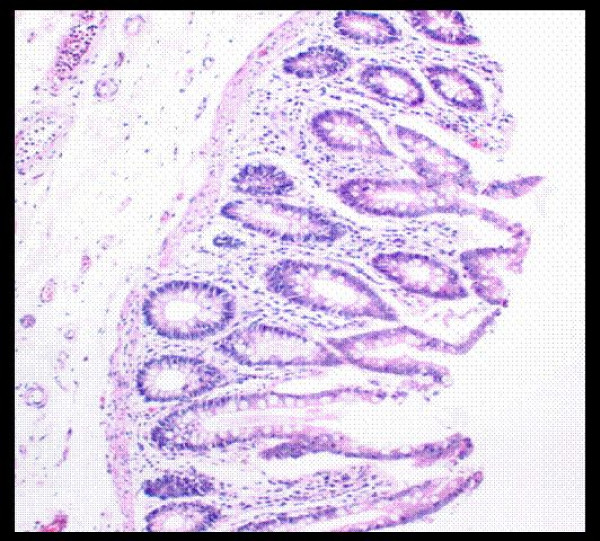
High-power view of mucosa showing architectural distortion and mild acute inflammation.

Microscopic examination of the specimen showed numerous elongated plicae (diaphragms) with submucosal fibrosis. In these areas the mucosa showed architectural distortion and mild acute inflammation (Figures 2 and 3). The background bowel showed mild architectural distortion and patchy acute inflammation within the lamina propria with possible focal cryptitis.

Review of the previous biopsies confirmed the histological diagnosis of active chronic inflammatory bowel disease in keeping with ulcerative colitis. Post-mortem examination concluded that stercoral perforation had led to sepsis and subsequent adult respiratory distress syndrome.

## Discussion

Diaphragm disease was first described by Lang et al. [1] in 1988 and has subsequently been mainly described in the literature as a rare side effect of NSAID usage. Many papers describe the occurrence of these diaphragms in the small bowel but only a few describe them in the large bowel. We therefore present this case as unusual, not only because the diaphragms occurred in the large bowel, but also as there was no apparent non-steroidal usage.

Diaphragm disease is a term used to describe the appearance of multiple (3 to 70), thin (2 to 4 mm) diaphragm-like strictures within the bowel [2]. These strictures can cause obstruction and subsequent perforation by narrowing the lumen to a pin-hole. The classical histological feature of a diaphragm is that of localised submucosal fibrosis, this feature distinguishes a diaphragm from exaggerated normal plicae in the bowel.

The pathogenesis of this condition is largely unknown although it has been suggested that damage to the mucosa, for example ulceration in any form, would cause fibrosis and scarring leading to contraction of the bowel wall and subsequent stenosis [3]. In this case, there was possible cause for ulceration in the form of inflammatory bowel disease but we could not elicit NSAID use after a full drug history and quizzing of the relatives. The general practitioner was also contacted; he had looked after her for 20 years and had only prescribed a short course of NSAIDs for dysmenorrhoea, which had been discontinued more than 5 years prior to presentation. Of course it is still possible that she had bought them over the counter, but from further discussions with the general practitioner he thought this was doubtful in this case.

Other causes of strictures include ischaemia, although this is unlikely in this case as she was relatively young and had no signs of being an arteriopath on post-mortem examination.

It is difficult to determine how long these diaphragms had been present as, although she had undergone an endoscopy in the past, it had never been possible to observe the proximal colon due to excessive pain. It is also difficult to definitely attribute the perforation to diaphragm disease as the majority of the disease was in the proximal large bowel. It is possible that the admission and perforation was due to refractory inflammatory bowel disease of the rectum and the diaphragm disease was an incidental finding. Although there were only mild microscopic signs of active inflammatory bowel disease in the resection specimen, previous biopsies from the stenosed areas had shown active disease and, therefore, the diaphragms may have been a result of previous ulceration in this area.

A further confounding factor in this case was the long-term high-dose immunosuppression. Given the presence of intraperitoneal gas on the radiograph, it may well be that the perforation had occurred several days previously and the delayed presentation with septic shock occurred as a result of the azathioprine and steroids masking the normal response.

## Conclusion

To the best of our knowledge, it would appear that this is the first reported case of diaphragm disease of the large bowel in the apparent absence of long-term NSAIDs. We would suggest that this supports the hypothesis put forward by Going et al. [3] in 1993 that diaphragm disease is the result of healing insults on the bowel mucosa, in this case in the form of inflammatory bowel disease.

## Abbreviations

NSAID: non-steroidal anti-inflammatory drug.

## Consent

Written informed consent could not be obtained in this case since the patient is deceased and the next-of-kin were untraceable. We believe this case report contains a worthwhile clinical lesson which could not be made as effectively in any other way. We expect the next-of-kin would not object to the publication of this case. All efforts have been made to ensure that the risk of identification of this patient has been minimised to prevent the identity of the patient being revealed, either to others or to the patient's relatives.

## Competing interests

The authors declare that they have no competing interests.

## Authors' contributions

MHL and GMS were responsible for the concept, JCR and GMS wrote the paper with specialist contributions from CC and GTW and the manuscript was reviewed and edited by GMS. All authors approved the final version.
